# Cancer-Specific Survival in Octogenarian Breast Cancer Patients: A Competing Risk Analysis of Prognostic Factors

**DOI:** 10.3390/curroncol33100636

**Published:** 2026-10-20

**Authors:** Özlem Mermut, Berrin Yalçın, Rıza Umar Gürsu, Esra Arslan, Bülent Akçe, Didem Can Trabulus, Semiha Battal Havare, Serap Karabıyık

**Affiliations:** 1 Department of Radiation Oncology, University of Health Sciences Turkey, Istanbul Training and Research Hospital, 34098 Istanbul, Turkey; 2 Department of Medical Oncology, University of Health Sciences Turkey, Istanbul Training and Research Hospital, 34098 Istanbul, Turkey; 3 Department of Nuclear Medicine, University of Health Sciences Turkey, Istanbul Training and Research Hospital, 34098 Istanbul, Turkey; 4 Department of General Surgery, University of Health Sciences Turkey, Istanbul Training and Research Hospital, 34098 Istanbul, Turkey; 5 Department of General Surgery, School of Medicine, Bahçeşehir University, 34734 Istanbul, Turkey; 6 Department of Pathology, University of Health Sciences Turkey, Istanbul Training and Research Hospital, 34098 Istanbul, Turkey; 7 Department of Radiology, University of Health Sciences Turkey, Istanbul Training and Research Hospital, 34098 Istanbul, Turkey

**Keywords:** breast cancer, octogenarians, competing risks, Fine–Gray model, Charlson Comorbidity Index, cancer-specific survival

## Abstract

Background: This study evaluated long-term survival outcomes in breast cancer patients aged ≥80 years, focusing on how prognostic factors change when competing mortality risks are considered. Methods: We retrospectively analyzed 106 octogenarian breast cancer patients treated between 2013 and 2023. Endocrine therapy was evaluated as a treatment-related prognostic factor. Treatment-related prognostic factors were evaluated using multivariable Cox proportional hazards and Fine–Gray competing-risks models. Results: The median age was 82 years, and the median follow-up time was 27.5 months (range, 6–99 months). In the multivariable Cox proportional hazards, biopsy compared with surgery (HR, 0.56; *p* = 0.037), presence of endocrine therapy (HR, 0.38; *p* = 0.003), and CCI ≥ 8 (HR, 9.96; *p* < 0.001) were independently associated with OS. In the Fine–Gray model, the associations of surgical procedure (*p* = 0.120) and endocrine therapy (*p* = 0.081) with breast cancer-specific mortality were no longer statistically significant. CCI ≥ 8 remained significantly associated with breast cancer-specific mortality (sHR, 3.76; 95% CI, 1.52–9.31; *p* = 0.004). Conclusions: In octogenarian breast cancer patients, surgery and endocrine therapy were associated with improved OS, but their associations with breast cancer-specific mortality were attenuated after accounting for competing risks. High CCI emerged as the most robust prognostic factor. These findings support incorporating comorbidity burden and competing-risks assessment into individualized treatment decisions in this population.

## Simple Summary

This study evaluated the impact of comorbidity burden, measured by the Charlson Comorbidity Index (CCI), on treatment and survival outcomes in 106 octogenarian breast cancer patients. Patients with a high comorbidity burden (CCI ≥ 8) had larger tumors, more advanced disease, and were less likely to undergo definitive surgery. Overall survival was significantly worse among patients with higher CCI scores, and high comorbidity burden remained independently associated with overall and breast cancer-specific mortality after multivariable and competing-risk analyses. These findings highlight the importance of considering comorbidity burden and competing mortality when individualizing treatment decisions in octogenarian patients with breast cancer.

## 1. Introduction

Advancing age is characterized by an increasing burden of comorbidities, reduced physiological reserve, and complex medication use. Malignancies have become increasingly prevalent in older populations, and breast cancer incidence rises substantially with advancing age, with its global burden expected to increase further in the coming decades [1,2]. Despite this demographic shift, older adults remain inadequately represented in clinical research, limiting the applicability of trial-based evidence to routine practice [3]. Consequently, treatment decisions in elderly patients with breast cancer require careful consideration of treatment benefit, toxicity, functional status, comorbidity burden, and competing health risks. In routine practice, therapeutic decision-making is therefore guided not by chronological age alone but by the overall health status and functional capacity of the patient [4].

Current international recommendations emphasize that treatment decisions in older women with breast cancer should integrate tumor characteristics and molecular subtype with geriatric assessment, life expectancy, comorbidities, functional status, and patient preferences [5]. Endocrine therapy remains an important component of treatment for hormone receptor-positive disease, while chemotherapy and HER2-directed therapy may be considered according to tumor biology, disease stage, expected benefit, and overall health status. In triple-negative disease, chemotherapy remains an important systemic treatment option for fit patients, whereas treatment intensity should be individualized in frail or highly comorbid patients. Thus, molecular subtype informs systemic treatment selection, but its application in octogenarians should be considered alongside geriatric and health-status assessment [5].

In octogenarians, non-cancer mortality may precede breast cancer-related death, creating a competing-risk framework that can challenge conventional survival analyses. Treating competing events as simple censoring may distort disease-specific survival estimates. Therefore, the present study aimed to evaluate prognostic determinants of survival in octogenarian patients with breast cancer using conventional survival and competing-risk analyses.

Presentation at Conference: A part of this study was previously presented as an oral presentation at the 15th Breast Diseases Congress held in Antalya/Turkey on (17-20/10/2019), under the title “Survival affecting factors in octogenarian breast cancer patients” (oral presentation no: OP 112). The abstract was published in the abstract book of the congress in European Journal of Breast Health (2019, Vol 15, pS36) [6]. At that time, the analysis included 34 patients. Since then, the cohort has been expanded to 106 patients with longer follow-up, and additional analyses, including competing risk evaluation, have been performed. The present manuscript has not been published in full elsewhere.

## 2. Materials and Methods

### 2.1. Study Population and Definitions

This study evaluated patients aged 80–89 years diagnosed with breast cancer who presented to our clinic between 2013 and 2023. The final follow-up was conducted in December 2023. Follow-up data were available for all patients, and no patients were lost to follow-up. Survival status and cause of death were ascertained through hospital records. Histopathological data were obtained from medical records. Estrogen receptor (ER) and progesterone receptor (PR) positivity were defined as ≥1% nuclear staining on immunohistochemistry, in accordance with ASCO/CAP guidelines [7]. HER2 status was determined based on standard immunohistochemical and/or in situ hybridization criteria [8]. The Ki-67 proliferation index was assessed by immunohistochemistry in hotspot areas, and a cutoff value of 20% was used to define high proliferative activity, based on the 2013 St Gallen International Expert Consensus recommendations [9]. Breast cancer subtypes were classified into five IHC-based surrogate subgroups using ER, PR, HER2, and Ki-67 status: Luminal A (ER-positive and/or PR-positive, HER2-negative, and Ki-67 ≤ 20%), Luminal B/HER2-negative (ER-positive and/or PR-positive, HER2-negative, and Ki-67 > 20%), Luminal B/HER2-positive (ER-positive and/or PR-positive, HER2-positive, regardless of Ki-67 level), HER2-enriched (ER-negative, PR-negative, and HER2-positive), and triple-negative breast cancer (TNBC; ER-negative, PR-negative, and HER2-negative) [9]. For survival analyses, Luminal A and Luminal B/HER2-negative tumors were combined into a single Luminal group, resulting in four molecular subtype groups: Luminal, Luminal B/HER2-positive, HER2-enriched, and TNBC.

The Charlson Comorbidity Index (CCI) was calculated for each patient [10] using the online MDCalc calculator. Given that all patients were aged ≥80 years, age contributed 4 points to the CCI score, while the diagnosis of non-metastatic breast cancer contributed an additional 2 points. Therefore, the minimum baseline CCI score was 6. Based on the distribution of the data and to ensure statistical robustness, patients were categorized into two groups: CCI 6–7 and CCI ≥ 8. Patients with CCI scores of 8, 9, and 10 were combined into the CCI ≥ 8 group because of the limited number of patients with individual scores of 8 and 9.

All patients were evaluated and discussed at the institutional multidisciplinary breast tumor board as part of the treatment planning process. Treatment strategies were individualized based on physical performance, cognitive status, comorbidities, and polypharmacy, with final treatment decisions made through shared decision-making with the patients and/or their families. Exclusion criteria included male sex, bilateral breast cancer, a prior history of other malignancies, ductal carcinoma in situ (DCIS) histology, and metastatic disease at the time of initial diagnosis. All included patients underwent initial staging with 18F-FDG positron emission tomography/computed tomography (PET/CT). using a Biograph mCT 20 ultraHD scanner (Siemens Molecular Imaging, Hoffman Estates, IL, USA).

### 2.2. Primary and Secondary Outcomes

The primary outcome of the study was overall survival (OS), defined as the time from breast cancer diagnosis to death from any cause. Patients who were alive at the last follow-up were censored at the date of their last known follow-up. Secondary outcomes included disease-free survival (DFS), breast cancer-free survival (BCFS), and breast cancer-specific survival (BCSS). DFS was defined as the time from diagnosis to the first occurrence of breast cancer recurrence, including local, regional, or distant recurrence, or death from any cause, whichever occurred first. BCFS was defined as the time from diagnosis to the first occurrence of breast cancer recurrence, including local, regional, or distant recurrence, or death from breast cancer, whichever occurred first. BCSS was defined as the time from diagnosis to death attributable to breast cancer, with deaths from other causes treated as competing events.

### 2.3. Statistical Analysis

Statistical analyses were performed using jamovi (Version 2.6.44; The jamovi Project, 2024) and R (Version 4.6.1; R Core Team, 2026). Fine–Gray competing-risks analyses were performed in R using the cmprsk package. Continuous variables were presented as mean ± standard deviation or median (minimum–maximum), as appropriate, and categorical variables as counts and percentages. Group comparisons were performed using the independent-samples *t*-test or Mann–Whitney *U* test for continuous variables and the chi-square test or Fisher’s exact test for categorical variables, as appropriate.

Overall survival (OS), disease-free survival (DFS), and breast cancer-free survival (BCFS) were estimated using the Kaplan–Meier method, and survival distributions were compared using the log-rank test. Survival analyses were performed in the overall study population and according to molecular subtype.

Multivariable Cox proportional hazards regression was used to identify independent prognostic factors. Covariates for the multivariable models were selected a priori based on clinical relevance and established prognostic importance, while considering the number of observed events to minimize the risk of over-parameterization. The final multivariable models included CCI category (6–7 vs. ≥8), surgical procedure (surgery vs. biopsy), and endocrine therapy (present vs. absent). Patient age was not included as an independent covariate because age is inherently incorporated into the calculation of the CCI. To minimize potential multicollinearity between hormone receptor status and endocrine therapy, only endocrine therapy was included as the treatment-related covariate. Tumor stage was considered clinically relevant and was evaluated in univariable analyses but was not retained in the final multivariable models because of the limited number of events and the need to maintain a parsimonious model.

The same prespecified covariates were subsequently evaluated using Fine–Gray competing-risks models for breast cancer-specific survival (BCSS), with breast cancer-related death defined as the event of interest and deaths from other causes treated as competing events. Given the limited number of breast cancer-specific events (*n* = 19), the final models were kept parsimonious to minimize over-parameterization. Hazard ratios (HRs) and subdistribution hazard ratios (sHRs), with corresponding 95% confidence intervals (CIs), were reported. A two-sided *p*-value < 0.05 was considered statistically significant.

## 3. Results

### 3.1. Baseline Characteristics and Treatment Patterns

A total of 106 octogenarian breast cancer patients were included in the study, of whom 77 (72.6%) had a CCI score of 6–7 and 29 (27.4%) had a CCI score of ≥8. The mean age was 82.9 ± 2.86 years in the CCI 6–7 group and 83.7 ± 3.23 years in the CCI ≥ 8 group, with no significant difference between the groups (*p* = 0.182). Patients with CCI ≥ 8 had significantly larger tumors than those with CCI 6–7 (median, 4.1 vs. 2.7 cm; *p* = 0.001).

The distribution of histological type, nuclear grade, estrogen receptor status, progesterone receptor status, HER2 status, and Ki-67 proliferation index did not differ significantly between the CCI groups. In contrast, disease stage differed significantly between the groups (*p* < 0.001), with stage III disease being more frequent among patients with CCI ≥ 8 (65.5% vs. 22.1%), whereas no patient in this group had stage I disease.

Surgical management also differed significantly according to CCI category (*p* = 0.007). Biopsy without definitive surgery was more frequent in patients with CCI ≥ 8 (58.6% vs. 28.6%), whereas breast-conserving surgery was performed only in the CCI 6–7 group. The distribution of IHC-based surrogate subtypes did not differ significantly between the CCI groups (*p* = 0.214).

Endocrine therapy and chemotherapy and/or HER2-targeted therapy use were comparable between the groups (*p* = 0.163 and *p* = 0.493, respectively). Radiotherapy patterns differed significantly between the CCI groups (*p* = 0.004), with palliative radiotherapy being more frequent among patients with CCI ≥ 8 (24.1% vs. 2.6%). Baseline characteristics and treatment patterns of octogenarian breast cancer patients stratified by CCI score are shown Table 1.

### 3.2. Survival Outcomes and Predictors of Mortality

The median follow-up period for the entire cohort of 106 octogenarian breast cancer patients was 27.5 months (IQR, 39.8 months, range, 6–99 months). The median overall survival (OS) was 46.0 months (95% CI, 39.0–60.0). During follow-up, 62 patients died, while 44 remained alive at the last follow-up. Overall, 19 patients died from breast cancer and 43 from non-cancer-related causes. In the CCI 6–7 group, 40 patients (51.9%) remained alive at the last follow-up, while 8 (10.4%) died from breast cancer and 29 (37.7%) from non-cancer-related causes. In the CCI ≥ 8 group, 4 patients (13.8%) remained alive, while 11 (37.9%) died from breast cancer and 14 (48.3%) from non-cancer-related causes.

During follow-up, the pattern of breast cancer recurrence or metastasis differed significantly between the CCI groups (*p* < 0.001). In the CCI 6–7 group, bone metastases occurred in 4 patients (5.2%), lung metastases in 2 (2.6%), brain metastases in 3 (3.9%), lymph node recurrence in 1 (1.3%), and local recurrence in 2 (2.6%). In the CCI ≥ 8 group, bone metastases occurred in 8 patients (27.6%), lung metastases in 1 (3.4%), brain metastases in 1 (3.4%), multiple metastatic sites in 5 (17.2%), and contralateral axillary involvement in 2 (6.9%). No recurrence or metastatic disease was observed during follow-up in 65 patients (84.4%) in the CCI 6–7 group compared with 12 patients (41.4%) in the CCI ≥ 8 group.

In univariable Cox regression analysis, higher CCI was strongly associated with poorer OS (HR, 10.26; 95% CI, 5.65–18.61; *p* < 0.001). Tumor diameter (HR, 1.73; 95% CI, 1.01–2.95; *p* = 0.044), stage III disease compared with stage I–II disease (HR, 2.34; 95% CI, 1.40–3.93; *p* = 0.001), and TNBC compared with the Luminal group (HR, 3.23; 95% CI, 1.58–6.62; *p* = 0.001) were also associated with poorer OS. Conversely, surgery compared with biopsy (HR, 0.44; 95% CI, 0.26–0.75; *p* = 0.003), ER positivity (HR, 0.39; 95% CI, 0.21–0.72; *p* = 0.003), PR positivity (HR, 0.40; 95% CI, 0.23–0.72; *p* = 0.002), and endocrine therapy (HR, 0.39; 95% CI, 0.21–0.74; *p* = 0.004) were associated with lower mortality. Detailed univariable Cox regression results are presented in Supplementary Table S1.

In the overall cohort, the 1-, 3-, and 5-year OS rates were 80.2%, 63.5%, and 36.2%, respectively. The corresponding rates for DFS were 55.2%, 38.8%, and 19.4%; for BCFS, 71.9%, 58.6%, and 34.6%; and for BCSS, 86.2%, 80.7%, and 78.4%, respectively (Supplementary Figure S1).

The CCI scores ranged from 6 to 10, with scores of 6, 7, 8, 9, and 10 observed in 17 (16.0%), 60 (56.6%), 4 (3.8%), 6 (5.7%), and 19 (17.9%) patients, respectively (Supplementary Table S2).

**Figure 1:**
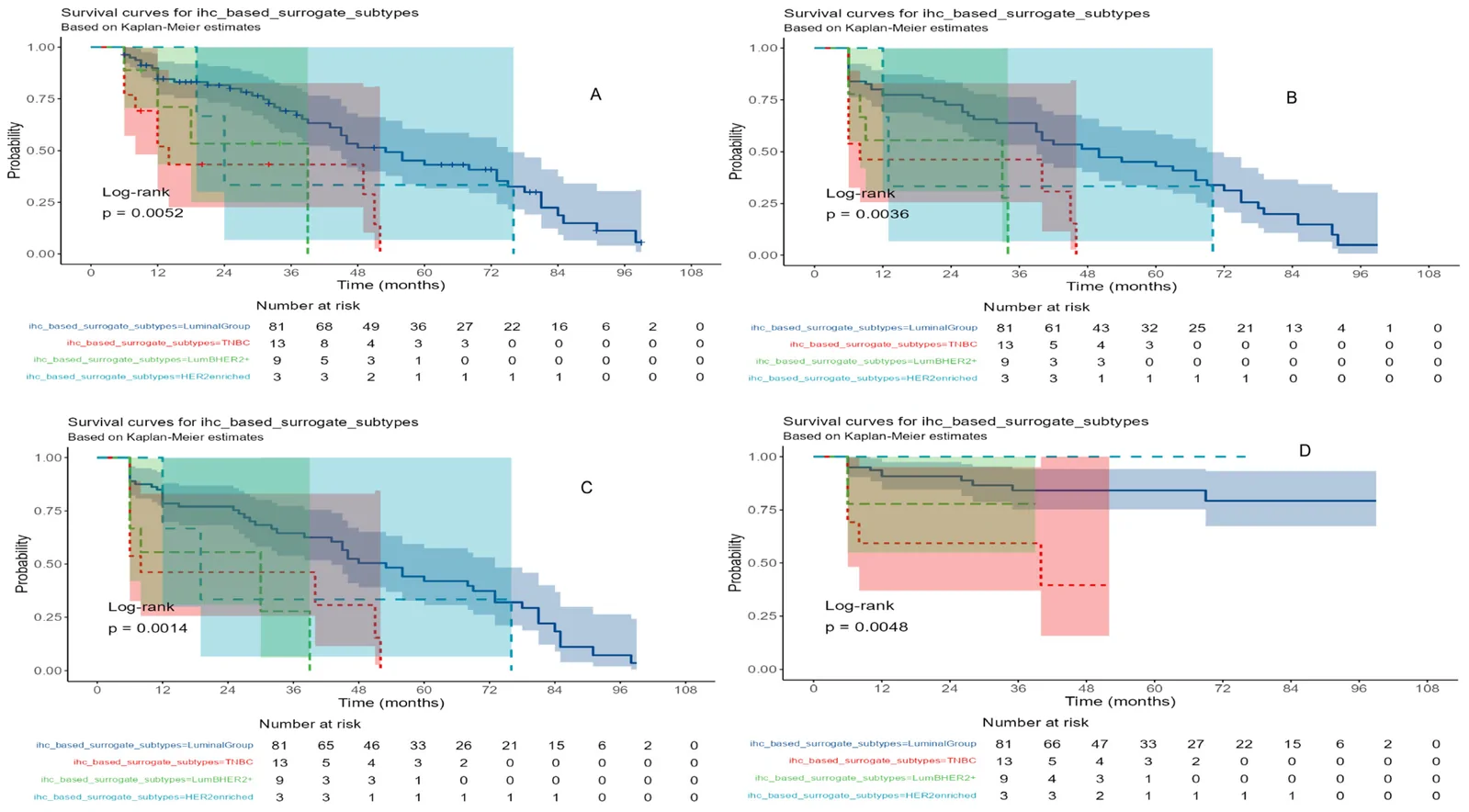
Kaplan–Meier survival curves according to IHC-based surrogate molecular subtypes. (**A**) Overall survival, (**B**) disease-free survival, (**C**) breast cancer-free survival, and (**D**) breast cancer-specific survival. Luminal A and Luminal B/HER2-negative tumors were combined into the Luminal group. *p*-values were calculated using the log-rank test. The Luminal group is represented by a solid dark blue line, TNBC by a dashed red line, Luminal B/HER2-positive by a dashed green line, and HER2-enriched by a dashed light blue line.

Kaplan–Meier analyses according to IHC-based surrogate subtypes showed no statistically significant difference in OS among the four subtype groups (log-rank *p* = 0.052). In contrast, DFS, BCFS, and BCSS differed significantly among the subgroups (log-rank *p* = 0.0036, *p* = 0.0014, and *p* = 0.0048, respectively). Kaplan–Meier curves for OS, DFS, BCFS, and BCSS according to IHC-based surrogate subtypes are presented in Figure 1.

In the multivariable Cox regression analysis, CCI ≥ 8 was strongly associated with increased mortality compared with CCI 6–7 (HR, 9.96; 95% CI, 5.37–18.48; *p* < 0.001). Endocrine treatment was associated with lower mortality (HR, 0.38; 95% CI, 0.20–0.72; *p* = 0.003), while biopsy was associated with a lower hazard of death compared with surgery (HR, 0.56; 95% CI, 0.32–0.97; *p* = 0.037) (Table 2).

### 3.3. Competing Risk Analysis of Breast Cancer-Specific Mortality

In the competing-risks analysis using the Fine–Gray model, the association between CCI ≥ 8 and breast cancer-specific mortality remained statistically significant, although the effect estimate was attenuated (sHR, 3.76; 95% CI, 1.52–9.31; *p* = 0.004). In contrast, neither endocrine treatment (sHR, 0.45; 95% CI, 0.14–1.11; *p* = 0.081) nor biopsy compared with surgery (sHR, 0.47; 95% CI, 0.18–1.22; *p* = 0.120) remained statistically significant after accounting for competing risks (Table 2).

**Figure 2:**
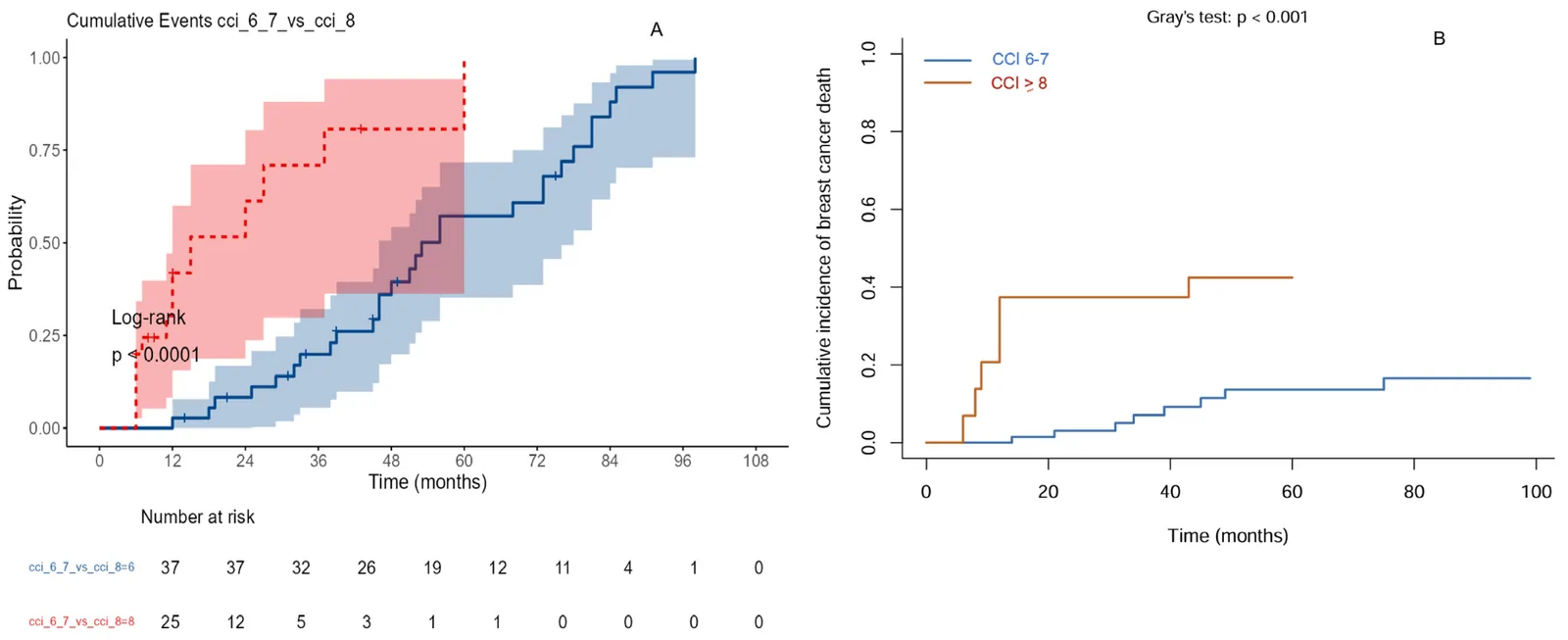
Competing-risk analysis of breast cancer-specific mortality according to CCI. (**A**) Cumulative incidence function. (**B**) Multivariable Fine–Gray regression analysis. Blue represents CCI scores of 6–7, and red represents a CCI score of 8.

The cumulative incidence of breast cancer-specific mortality at 12, 36, and 60 months was 0.0% (95% CI, 0.0–0.0%), 7.1% (95% CI, 0.3–13.9%), and 13.6% (95% CI, 4.0–23.3%), respectively, in the CCI 6–7 group, compared with 37.4% (95% CI, 18.5–56.4%), 37.4% (95% CI, 18.5–56.4%), and 42.5% (95% CI, 21.6–63.3%), respectively, in the CCI ≥ 8 group. The cumulative incidence functions, with non-cancer deaths treated as competing events, are shown in Figure 2A, together with the multivariable Fine–Gray regression analysis in Figure 2B.

## 4. Discussion

Our findings indicate that conventional survival analyses may overestimate the apparent survival benefits associated with cancer-directed treatments in octogenarian breast cancer patients when competing non-cancer mortality is not considered. In our cohort, surgery and endocrine therapy were associated with overall survival in conventional Cox regression, whereas these associations were attenuated after accounting for competing mortality using the Fine–Gray model. Although biopsy was associated with a lower hazard of death than surgery in the conventional Cox model, this association was no longer statistically significant after competing-risk adjustment. This finding should be interpreted cautiously, as the choice of definitive surgery was likely influenced by baseline health status, comorbidity burden, disease extent, and treatment preferences. In contrast, comorbidity burden, measured by the Charlson Comorbidity Index (CCI), remained strongly associated with both overall and breast cancer-specific outcomes. These findings highlight the importance of considering competing risks when evaluating treatment outcomes in the oldest-old population and represent an important contribution of the present study to the existing literature. The present study adds to the existing literature by demonstrating, in a cohort of octogenarian breast cancer patients with long-term follow-up, how accounting for competing non-cancer mortality can substantially alter the interpretation of treatment-associated survival outcomes.

Breast cancer in octogenarians differs from that in younger patients because treatment decisions are influenced not only by tumor characteristics but also by comorbidity, functional status, frailty, and life expectancy. In our cohort, invasive ductal carcinoma was the predominant histologic subtype, consistent with previous reports [10,11,12,13,14]. Treatment patterns in older patients are generally selective and individualized; in our cohort, radiotherapy was administered to 53.8% of patients, consistent with patterns reported in previous studies of older breast cancer patients [11,12,13,14,15]. Although tumor stage, nuclear grade, and tumor size showed associations with outcome in univariable analyses, these established clinicopathological factors have well-recognized prognostic relevance in breast cancer [16,17]. In older patients, the potential benefit of systemic treatment may also vary according to disease stage, tumor biology, and patient characteristics [18,19]. Rather than focusing extensively on these established prognostic variables, our findings suggest that their interpretation in octogenarians should be integrated with geriatric characteristics and competing mortality.

The most important finding of our study was the strong prognostic role of comorbidity. A high CCI score (≥8) independently predicted overall survival in the multivariable Cox model and was independently associated with a higher cumulative incidence of breast cancer-specific mortality in the Fine–Gray model. Previous population-based evidence has demonstrated that comorbidity is strongly associated with other-cause mortality in older patients with breast cancer, supporting the incorporation of comorbidity and competing mortality into individualized treatment assessment [20]. In contrast to reports in which breast cancer-specific mortality predominates during the first five years [21], our findings highlight the substantial contribution of non-cancer mortality among octogenarians with a high comorbidity burden. Previous studies have also suggested that surgery and systemic treatment may provide survival benefits in selected older patients with breast cancer [22,23]. However, these benefits may be influenced by age, comorbidity burden, and competing mortality risks. Our findings extend these observations by demonstrating that the apparent survival benefits associated with surgery and endocrine therapy in conventional Cox analysis were attenuated after accounting for competing risks, whereas high CCI remained independently associated with breast cancer-specific mortality. Importantly, CCI alone does not capture the multidimensional health status of older patients. Similarly, conventional performance status measures may fail to identify clinically relevant deficits in functional status, cognition, nutrition, and other geriatric domains, even among patients considered to have good performance status. Comprehensive geriatric assessment can identify these additional vulnerabilities and may help guide treatment intensity and supportive care in older adults. Recent evidence also suggests that integrating comprehensive geriatric assessment with patient coaching can improve quality of life in older patients receiving systemic therapy [24], while frailty assessments remain underused in older women with breast cancer [25]. Therefore, CCI should ideally be complemented by performance status and comprehensive geriatric assessment, including functional status, frailty, and quality-of-life considerations, when individualizing treatment for octogenarian patients [26].

Systemic treatment should likewise be individualized according to tumor biology and geriatric status. Endocrine therapy was administered to 84% of our patients, reflecting the high prevalence of hormone receptor-positive disease in older breast cancer populations [12,13,14]. Consistent with previous reports [12,27], endocrine therapy was associated with improved overall survival in our conventional analysis, although this association was not maintained in the Fine–Gray competing-risk model. Chemotherapy was administered to 21 patients (19.8%), consistent with the lower use of systemic chemotherapy reported in elderly populations [11,12,19]. Previous evidence suggests that the potential benefit of chemotherapy may differ according to molecular subtype; for example, Yamada et al. reported improved overall survival among selected luminal and HER2-positive patients receiving chemotherapy [18]. In our study, molecular subtypes were additionally evaluated in survival and regression analyses. However, the small number of patients in some subgroups, particularly the HER2-enriched group, resulted in limited precision and unstable estimates in multivariable analyses. These findings support consideration of molecular subtype when assessing systemic treatment, while emphasizing that treatment decisions in octogenarians should also incorporate comorbidity, functional status, life expectancy, and treatment tolerance [28]. The metastatic pattern observed in our cohort, including the predominance of bone involvement and frequent pulmonary involvement, was broadly consistent with previous observations in octogenarian patients [29].

Our findings also have important implications for treatment selection and interpretation of outcomes in the oldest old. Schonberg et al. demonstrated that women aged ≥80 years may receive less intensive treatment despite having tumor characteristics comparable to younger patients and may experience poorer survival outcomes [30]. However, conventional survival analyses may not adequately distinguish cancer-related benefit from the effect of competing mortality. In our cohort, the attenuation of the associations of surgery and endocrine therapy after competing-risk adjustment suggests that treatment effects should be interpreted in the context of baseline health status and competing causes of death. Similarly, the substantial burden of non-cancer mortality observed among patients with high CCI scores reinforces the importance of considering comorbidity, functional status, and life expectancy when determining treatment intensity [20,25,28]. A patient-centered approach does not imply withholding effective cancer treatment; rather, it supports matching the intensity of treatment to the patient’s overall health status, expected benefit, treatment tolerance, and preferences.

The primary strength of this study is the relatively long follow-up period, with a maximum follow-up of 99 months, together with the use of Fine–Gray competing-risk analysis, allowing a more appropriate assessment of breast cancer-specific outcomes in the presence of substantial non-cancer mortality. Nevertheless, several limitations should be acknowledged. This was a retrospective, single-center study with a relatively small sample size, limiting statistical power and precision, particularly within molecular subgroups. The limited number of breast cancer-specific deaths (*n* = 19) further restricted the number of variables that could be reliably included in the competing-risks model. Although disease stage and molecular subtype were evaluated in the baseline and univariable analyses, they were not included in the primary multivariable models because of concerns regarding overfitting and the reliability of estimates. Additional models incorporating molecular subtype alongside endocrine treatment produced unstable estimates and were therefore not considered reliable for interpretation. Consequently, residual confounding related to tumor characteristics, particularly hormone receptor status, and treatment selection cannot be excluded, and the observed association between endocrine therapy and overall survival should not be interpreted as evidence of a causal treatment benefit. Although all cases were reviewed by a multidisciplinary breast council and treatment decisions were individualized, no formal geriatric assessment was performed. Consequently, standardized measures of frailty, functional status, performance status, quality of life, and treatment adherence were unavailable and may have influenced treatment selection and outcomes. Despite these limitations, our findings emphasize that baseline health status and competing mortality are important considerations in octogenarian breast cancer. As life expectancy increases, treatment decisions in these patients should integrate tumor characteristics and molecular subtype with comorbidity, functional status, frailty, quality of life, life expectancy, and patient preferences to support individualized treatment and appropriately balance treatment benefits and burdens. Larger multicenter studies with adequate event numbers and comprehensive tumor and geriatric assessments are needed to validate these findings.

## Figures and Tables

**Table 1:** Baseline characteristics and treatment patterns of octogenarian breast cancer patients stratified by CCI score.

	CCI 6–7 *n*: 77 (%)	CCI ≥ 8 *n*: 29 (%)	*p* Value
Age (mean ± SD) Median (min-max)	82.9 ± 2.86 82 (80–89)	83.7 ± 3.23 83 (80–89)	0.182 ^t^
Tumor diameter (mean ± SD) Median (min-max)	3.2 ± 1.75 2.7 (1–9.8)	4.63 ± 2.6 4.1 (2–14.1)	0.001 ^m^
Histology			
Invasive ductal cancer	57 (74)	22 (75.9)	
Others	20 (26)	7 (24.1)	0.847
Surgical procedure			
Mastectomy (SLNB and/or AD)	46 (59.7)	12 (41.4)	
Breast-conserving surgery (SLNB and/or AD)	9 (11.7)	0 (0)	
Biopsy	22 (28.6)	17 (58.6)	0.007 ^f^
Nuclear grade			
2	40 (51.9)	14 (48.3)	
3	37 (48.1)	15 (51.7)	0.736
Stages			
I	15 (19.5)	0 (0)	
II	45 (58.4)	10 (34.5)	
III	17 (22.1)	19 (65.5)	<0.001 ^f^
Estrogen receptor			
positive	68 (88.3)	22 (75.9)	
negative	9 (11.7)	7 (24.1)	0.110
Progesterone receptor			
positive	57 (74)	9 (31)	
negative	20 (26)	20 (69)	0.602
Her2 status			
positive	7 (9.1)	5 (17.2)	
negative	70 (90.9)	24 (82.8)	0.238
IHC-based surrogate subtypes			
LuminalA	36 (46.8)	12 (41.4)	
LuminalB-HER2-	27 (35.1)	6 (20.7)	
LuminalB-HER+	5 (6.5)	4 (13.8)	
HER2 enriched	2 (2.6)	1 (3.4)	
Triple negative	7 (9.1)	6 (20.7)	0.214 ^f^
Endocrine treatment			
present	67 (87)	22 (75.9)	
absent	10 (13)	7 (24.1)	0.163
Chemotherapy and/or HER2 targeted therapy			
present	14 (18.2)	7 (24.1)	
absent	63 (81.8)	22 (75.9)	0.493
Radiotherapy			
chest wall ± lymph nodes	36 (46.8)	12 (41.4)	
palliative (e.g., bone)	2 (2.6)	7 (24.1)	
absent	39 (50.6)	10 (34.5)	0.004 ^f^
Ki-67% ratio (mean ± SD) Median (min-max)	28.1 ± 19.1 23 (5–80)	31.4 ± 21.6 28 (10–80)	0.520 ^m^

(SLNB = Sentinel Lymph Node Biopsy, AD = Axillary Dissection, CCI = Charlson Comorbidity Index, t = samples *t*-test, f = Fisher’s exact test, m = Mann–Whitney U test).

**Table 2:** Comparison of multivariable Cox regression and Fine–Gray competing-risks regression analyses.

		Multivariable Cox Regression			Fine–Gray	
	HR	95% CI	*p* Value	sHR	95% CI	*p* Value
Surgical procedure						
surgery (ref)	1			1		
biopsy	0.56	0.32–0.97	0.037	0.47	0.18–1.22	0.120
Endocrine treatment						
absent (ref)	1			1		
present	0.38	0.20–0.72	0.003	0.45	0.14–1.11	0.081
CCI scores						
6–7 (ref)	1			1		
≥8	9.96	5.37–18.48	<0.001	3.76	1.52–9.31	0.004

## Data Availability

The data that support the findings of this study are not publicly available due to privacy restrictions but are available from the corresponding author upon reasonable request.

## Supplementary Materials

The following supporting information can be downloaded at: https://www.mdpi.com/article/10.3390/curroncol33100636/s1, Figure S1: Kaplan–Meier survival curves for OS, DFS, BCFS, BCSS; Table S1: Univariate Cox regression analysis all groups; Table S2: Distribution of Charlson Comorbidity Index (CCI) scores in the study population.

## Abbreviations

The following abbreviations are used in this manuscript:

CCI	Charlson Comorbidity Index
ER	Estrogen Receptor
PR	Progesterone Receptor
HER2	Human Epidermal Growth Factor Receptor 2
HR	Hazard Ratio
sHR	Subdistribution Hazard Ratio
CI	Confidence Interval
OS	Overall Survival
