# Supplementary material for: Cancer-Specific Survival in Octogenarian Breast Cancer Patients: A Competing Risk Analysis of Prognostic Factors
Source: Curr Oncol. 2026 Oct 20;33(10):636. doi: 10.3390/curroncol33100636 (PMC13702921; doi:10.3390/curroncol33100636)
Supplement: Supplementary file 1 [file curroncol-33-00636-s001.zip › Supplementary Table S1.Univariate Cox analyses.pdf]

Supplementary Table S1. Univariate Cox regression analysis all groups.

**Variables**	**HR**	**95 CI %**	***p* value**
Age	1.05	0.96-1.15	0.244
Histology<br>(IDC vs others)	0.65	0.34-1.22	0.177
Tumor diameter	1.73	1.01-2.95	0.044
Surgical procedure<br>surgery vs biopsy	0.44	0.26-0.75	0.003
Nuclear grade<br>2 vs 3	0.81	0.47-1.39	0.440
Stages<br>I-II vs III	2.34	1.40-3.93	0.001
ER status<br>negative vs positive	0.39	0.21-0.72	0.003
PR status<br>negative vs positive	0.40	0.23-0.72	0.002
Her2 status<br>negative vs positive	1.82	0.82-4.06	0.143
IHC-based surrogate subtypes<br>Luminal group (ref)	1	-	-
Triple negative	3.23	1.58-6.62	0.001
LuminalBHER2+	2.41	0.83-6.96	0.104
HER2 enriched	1.90	0.59-6.19	0.284
Endocrine treatment<br>present vs absent	0.39	0.21-0.74	0.004
Chemotherapy and/or targeted therapy<br>absent vs present	1.27	0.65-2.47	0.479
Radiotherapy<br>absent vs present	1.14	0.66-1.98	0.640
Ki-67 %<br><20 vs ≥20	0.99	0.60-1.65	0.970
CCI<br>≥8 vs 6-7	10.26	5.65-18.61	<0.001
