# Supplementary material for: Cancer-Specific Survival in Octogenarian Breast Cancer Patients: A Competing Risk Analysis of Prognostic Factors
Source: Curr Oncol. 2026 Oct 20;33(10):636. doi: 10.3390/curroncol33100636 (PMC13702921; doi:10.3390/curroncol33100636)
Supplement: Supplementary file 1 [file curroncol-33-00636-s001.zip › Supplementary Table S2.CCI scores.pdf]

Supplementary Table S2. Distribution of Charlson Comorbidity Index (CCI) scores in the study population.

**CCI scores**	**Number of patients (%)**
6	17 (16.0)
7	60 (56.6)
8	4 (3.8)
9	6 (5.7)
10	19 (17.9)
